# Surgery of the Vermiform Appendix

**Published:** 1906-01-20

**Authors:** 


### SURGERY OF THE VERMIFORM APPENDIX.

**Appendicitis.**—According to Sherren [1] the death-rate of the cases of acute appendicitis treated at the London Hospital during the years 1892 to 1905 inclusive varied between 17 and 23 per cent., with a mean of 19½ per cent. This fact suggests that some alteration in our treatment is desirable. Inflammation of the appendix is caused by the action of micro-organisms on a vulnerable appendix. It was formerly taught that the offending micro-organism was the colon bacillus, but opinions have lately undergone a change, and observers have pointed out that other organisms (staphylococcus, pneumococcus, etc.) are often present, and probably produce the mischief. Interference with the blood supply of the appendix is the most common cause of its increased vulnerability or lowered resistance, and is due in many cases to increased tension within the appendix. In acute appendicitis the increased tension is due in most cases to obstruction of the opening of the appendix into the cæcum. This orifice is small, and guarded in from 30 to 40 per cent. of cases by a valvular fold of mucous membrane, the valve of Gerlach. Swelling of the mucous membrane at this opening, such as may be produced in typhlitis, or obstruction, the result of distension of the cæcum, is the cause of the increased tension in many cases. But obstruction of the opening of the appendix into the cæcum is not the only way by which the pressure in the appendix may be raised. The obstruction may occur at a stricture, left after a previous attack, or produced as the result of chronic appendicitis. This stricture may become completely closed by swelling of mucous membrane, and, if completely closed, infection of the contents of the appendix distal to the stricture may precipitate an attack. Sherren states that he has found that operation, while cutaneous tenderness is present, invariably reveals a distended appendix. Some cases of inflammation of the appendix do not appear to be caused by interference with its blood supply as the result of increased tension, but arise in connection with injury. Sherren considers that the only way in which the death-rate can be reduced is by early operation. That a large percentage of cases get over the attack without surgical aid is true, but a large number of these cases relapse. All definite cases of appendicitis should be treated by removal of the appendix; the difficulty arises in deciding when this should be done. If we are able to see a case within the first 24 or 36 hours, when the disease is probably limited to the appendix, operation should be performed and the appendix removed. Often drainage is unnecessary, and the abdomen can be opened in such a way as to avoid the occurrence of a ventral hernia. If this were the routine treatment the death-rate should not be more than 2 per cent. After the most favourable time has passed the patient should, if possible, be tided over the attack, and the appendix removed later; but operation must be performed at any stage if special indications exist. All cases that are not markedly improving should be operated upon, no matter what stage of the disease they are in. The condition of the superficial tenderness is helpful in deciding the question of operation. This superficial tenderness is brought out by gently stroking or picking up the skin. It usually occupies an area named by Sherren "the appendix triangle"—bounded below by Poupart's ligament, above by a line drawn out from the umbilicus, and to the inner side by a vertical line just to the right of the mid-line. Its apex is at the anterior superior iliac spine. In cases that are quieting down, the triangle often becomes smaller and smaller, sometimes leaving a rounded area of tenderness midway between the anterior superior spine and the umbilicus. This superficial tenderness is due to stimulation of the nerves in the appendix, the result of the tension within it, and is a valuable sign that the appendix is not gangrenous or perforated. Disappearance of superficial tenderness, without evidence of improvement in the general condition of the patient, calls for immediate operation. In the treatment of appendicitis Morris [2] tells us that his views have altered materially since his first planned operation for removal of the appendix in 1902. He no longer uses long incisions, for it was noted that short incision patients started off more quickly towards comfortable recovery. Gauze packing has been discarded altogether, on the ground that it is a foreign body, that it causes ileus by direct mechanical effect, and excessive lymph formation, which leads to troublesome adhesions. Shock is caused by removal of gauze packing, and a serious defect is left in the abdominal wall in spite of provisional sutures. He uses a cigarette drain in all cases in which pus or septic debris have been left in the peritoneal cavity. Adhesions re-form at separated points in acute infectious cases, so that nothing is gained by separating them, but it does no harm to open freely into the normal peritoneal cavity in cases in which one is hunting for the appendix among adhesions. These conclusions are now agreed to by a large number of surgeons.

[1] Practitioner, June 1905, p. 833. [2] Med. Rec., May 27, 1905, p. 810.

